# In Situ Synthesis of All-Solid-State Z-Scheme BiOBr_0.3_I_0.7_/Ag/AgI Photocatalysts with Enhanced Photocatalytic Activity Under Visible Light Irradiation

**DOI:** 10.1186/s11671-018-2778-9

**Published:** 2018-11-20

**Authors:** Junlin Lu, Chaoqun Shang, Qingguo Meng, Haiqin Lv, Zhihong Chen, Hua Liao, Ming Li, Yongguang Zhang, Mingliang Jin, Mingzhe Yuan, Xin Wang, Guofu Zhou

**Affiliations:** 1 South China Academy of Advanced Optoelectronics, South China Normal University, Guangzhou, Guangdong, Province China; 2 Shenyang Institute of Automation, Chinese Academy of Sciences, Guangzhou, 511458 China; 3 International Academy of Optoelectronics, South China Normal University, Zhaoqing, Guangdong Province China; 4 Institute of Solar Energy, Yunnan Normal University, Kunming, 650500 People’s Republic of China

**Keywords:** Z-scheme, Photocatalytic, BiOBr_0.3_I_0.7_/Ag/AgI

## Abstract

A series of novel visible light driven all-solid-state Z-scheme BiOBr_0.3_I_0.7_/Ag/AgI photocatalysts were synthesized by facile in situ precipitation and photo-reduction methods. Under visible light irradiation, the BiOBr_0.3_I_0.7_/Ag/AgI samples exhibited enhanced photocatalytic activity compared to BiOBr_0.3_I_0.7_ and AgI in the degradation of methyl orange (MO). The optimal ratio of added elemental Ag was 15%, which degraded 89% of MO within 20 min. The enhanced photocatalytic activity of BiOBr_0.3_I_0.7_/Ag/AgI can be ascribed to the efficient separation of photo-generated electron–hole pairs through a Z-scheme charge-carrier migration pathway, in which Ag nanoparticles act as electron mediators. The mechanism study indicated that ·O_2_^−^ and h^+^ are active radicals for photocatalytic degradation and that a small amount of ·OH also participates in the photocatalytic degradation process.

## Background

To face critical energy and environmental crises, photocatalysis provides a potential strategy to solve these problems because it not only directly converts solar energy into chemical energy [1–6] but also degrades organic pollutants under solar light irradiation [7–12]. BiOBr_*x*_I_1−*x*_ solid solution, a novel BiOX-based photocatalyst, has drawn increasing interest because of its unique layered structure with special electrical and catalytic properties as well as its tunable band structure. However, BiOBr_*x*_I_1−*x*_ has a positive conduction band potential, which makes it exhibit a weak redox capability and restricts it from further application [13, 14].

Recently, it has been proved that Z-scheme photocatalytic systems are able to efficiently enhance the photocatalytic activity of semiconductors due to their special charge-carrier migration pathway [15–17]. In a typical Z-scheme photocatalytic system, the photo-generated hole and electron with approximate potential in different semiconductors will combine through an electron mediator. Thus, the photo-generated hole with more positive potential and the photo-generated electron with more negative potential can be maintained, which provides better redox ability. At the beginning, the electron mediator in Z-scheme systems was a shuttle redox mediator, such as I^−^/IO^3−^ and Fe^2+^/Fe^3+^ [15]; this kind of system is called PS-A/D-PS system. However, this kind of electron mediator is unstable for long-term use, and it has low electron transfer efficiency. In order to overcome this shortcoming, an all-solid-state Z-scheme system with noble metal as the electron mediator was developed [18–24]. With noble metal as the electron mediator, the charge transfer and the separation of photo-generated electron–hole pairs are more efficient, showing higher potential in practical applications. More recently, silver iodide has been widely applied in the construction of Z-scheme photocatalytic systems as the reductant due to its particular photolysis characteristics and negative conduction band potential. The in situ precipitation method provides a facile route for synthesizing silver iodide on the substrate content I element. Furthermore, Ag nanoparticles could be in situ photo-reduced by AgI to construct a Z-scheme photocatalytic system such as Ag_3_PO_4_/AgI [21], AgI/Ag/AgBr [22], AgI/Ag/Bi_2_MoO_6_ [23], or AgI/Ag/I-(BiO)_2_CO_3_ [24]. Thus, using an in situ precipitation and photo-reduction method to construct a novel Z-scheme photocatalyst between BiOBr_*x*_I_1−*x*_ and AgI would be a possible strategy to enhance the photocatalytic activity and the redox capability of BiOBr_*x*_I_1−*x*_.

In this study, all-solid-state Z-scheme BiOBr_0.3_I_0.7_/Ag/AgI photocatalysts with different mole ratios of elemental Ag are synthesized via in situ precipitation and photo-reduction methods. The BiOBr_0.3_I_0.7_/Ag/AgI photocatalysts are characterized by various technologies. The photocatalytic activities are evaluated by the degradation of methyl orange (MO) under visible light irradiation, and the optimal molar fraction of elemental Ag in the photocatalyst is determined. In addition, the photocatalytic mechanism is also investigated.

## Methods/Experimental

### Materials

Bismuth nitrate pentahydrate (Bi(NO_3_)_3_·5H_2_O), tertiary butanol (t-BuOH), and silver nitrate (AgNO_3_) were purchased from Aladdin Industrial Corporation. Potassium bromide (KBr), potassium iodide (KI), and methyl orange (MO) were purchased from Tianjin Zhiyuan Chemical Co., Ltd. All reagents were used without further purification.

### Synthesis of BiOBr_0.3_I_0.7_, BiOBr_0.3_I_0.7_/AgI, and BiOBr_0.3_I_0.7_/Ag/AgI

BiOBr_0.3_I_0.7_ solid solution was prepared by the ultrasound-assisted hydrolysis method according to a previous report [13]. BiOBr_0.3_I_0.7_/AgI photocatalyst was synthesized by the in situ precipitation method. A total of 0.5 g of BiOBr_0.3_I_0.7_ was added to 50 ml of appropriate concentration of AgNO_3_ solution, where the molar ratio of Ag to I was 15%. Then, the suspension was stirred at room temperature for 1 h to precipitate AgI, and the obtained sample was centrifuged, washed three times with deionized water, and dried at 60 °C for 12 h.

A series of BiOBr_0.3_I_0.7_/Ag/AgI photocatalysts were obtained by in situ precipitation and photo-reduction methods. In a typical experiment, 0.5 g of BiOBr_0.3_I_0.7_ was added to 50 ml of different concentrations of AgNO_3_ solutions, and the suspensions were stirred at room temperature for 1 h to precipitate AgI. Next, the obtained suspensions were irradiated with a 300 W Xe lamp (200 mW/cm^2^) for 10 min with continuous stirring to photo-reduce Ag nanoparticles. Finally, the obtained samples were centrifuged, washed three times with deionized water and dried at 60 °C for 12 h. By changing the AgNO_3_ concentration, BiOBr_0.3_I_0.7_/Ag/AgI with different Ag/I molar ratios was prepared. When the Ag/I molar ratios were 5%, 10%, 15%, and 20%, the as-prepared samples were named BAA-1, BAA-2, BAA-3, and BAA-4, respectively.

### Material Characterization

The crystal structures of the prepared photocatalysts were characterized on a Bruker D8 ADVANCE X-ray diffraction (XRD) instrument. Scanning electron microscopy (SEM) images and energy-dispersive spectroscopy (EDS) data were recorded on a Zeiss Ultra 55 thermal FESEM system. Transmission electron microscopy (TEM) and high-resolution TEM (HRTEM) images were recorded on a JEM-2100 instrument. X-ray photoelectron spectroscopy (XPS) measurements were carried out on a Thermo ESCALAB 250Xi instrument with a monochromatized Al Ka line source (150 W). Electron paramagnetic resonance (EPR) measurements were carried out on a Bruker ER 200-SRC spectrometer. UV–visible diffuse reflectance spectroscopy (UV–Vis DRS) measurements were conducted on a U-41000 HITACHI spectrophotometer (Tokyo, Japan) using BaSO_4_ as the reference.

### Photocatalytic Activity Tests

The photocatalytic activity of the prepared photocatalyst was determined by the degradation of MO under visible-light irradiation. In a typical experiment, a 300-W Xe lamp (AM 1.5, output light current of 15 A, 200 mW/cm^2^) with a 400-nm cut-off filter was used as the visible-light source, and the overall system was cooled by circulating water. A total of 100 mg of the prepared photocatalyst was added to 150 mL of an aqueous solution containing 10 mg/L MO. Then, the suspension was stirred for 30 min in the dark to attain adsorption–desorption equilibrium. After that, the suspension was irradiated using a Xe lamp, and 7 mL of the solution was sampled and centrifuged to remove the catalysts at 5 min time intervals. The concentration of MO in the degraded solution was detected by UV–vis spectroscopy at 465 nm.

## Results and Discussion

### Structure and Morphology Analysis

**Fig. 1:**
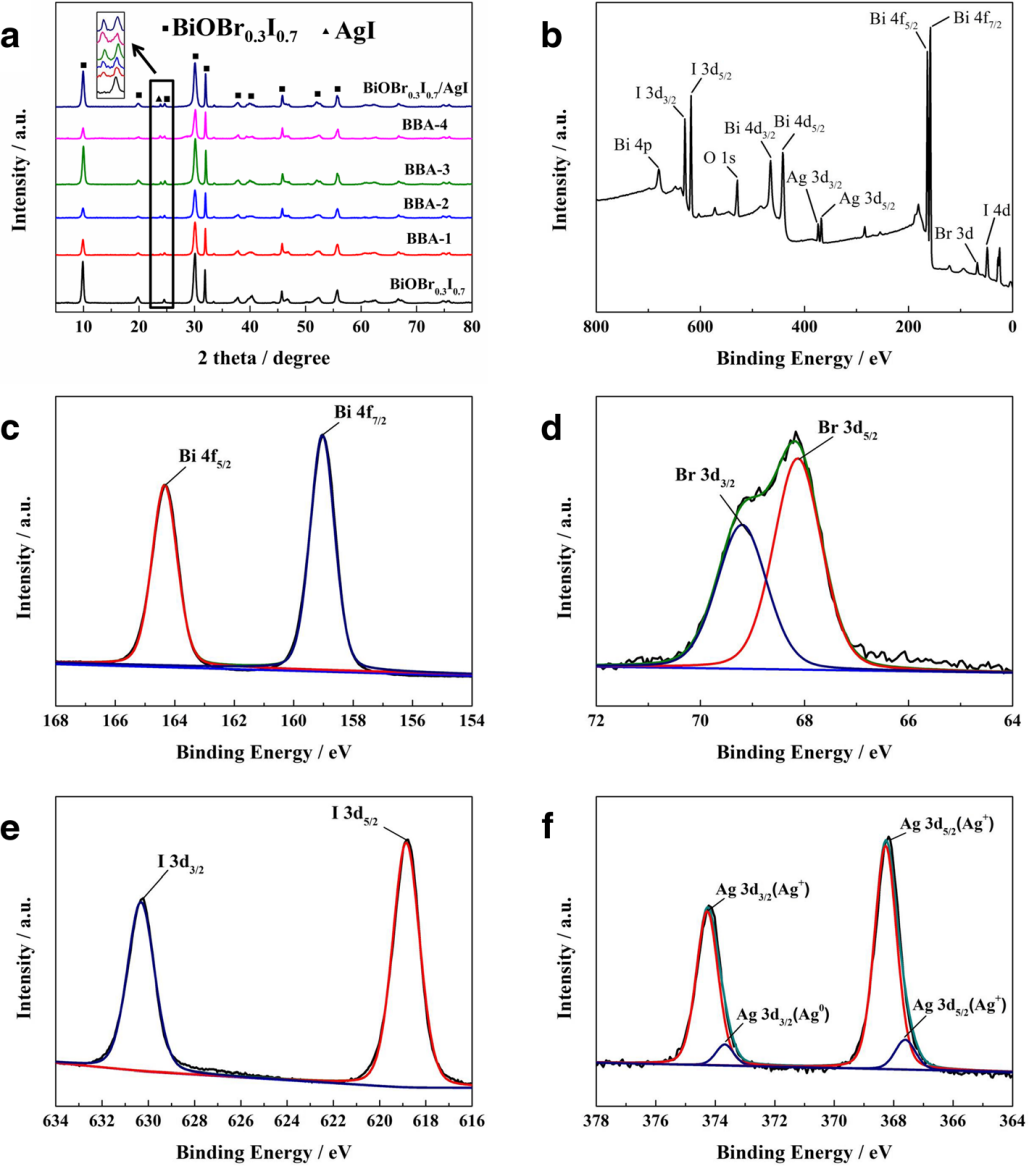
XRD patterns of BiOBr_0.3_I_0.7,_ BAA-x and BiOBr_0.3_I_0.7_/AgI (**a**), XPS survey spectra of BAA-15 (**b**), BAA-15 XPS spectra of Bi 4f (**c**), Br 3d (**d**), I 3d (**e**), and Ag 3d (**f**)

The XRD patterns of BiOBr_0.3_I_0.7_, BAA-*x* and BiOBr_0.3_I_0.7_/AgI are shown in Fig. 1a. All the diffraction peaks of BiOBr_0.3_I_0.7_ can be found in BAA-*x* and BiOBr_0.3_I_0.7_/AgI, which indicates that the construction of the Z-scheme photocatalytic system did not change the crystal phase of BiOBr_0.3_I_0.7_. The diffraction peak around 23.7 degrees belongs to AgI (JCPDS No. 09-0399), corresponding to the (111) diffraction. With an increasing elemental Ag ratio, the intensity of the diffraction peak of BAA-*x* at 23.7 degrees is increased, indicating that the AgI is deposited on the BBA-*x* surface. In addition, no diffraction peaks for Ag^0^ were found, due to its low content.

**Fig. 2:**
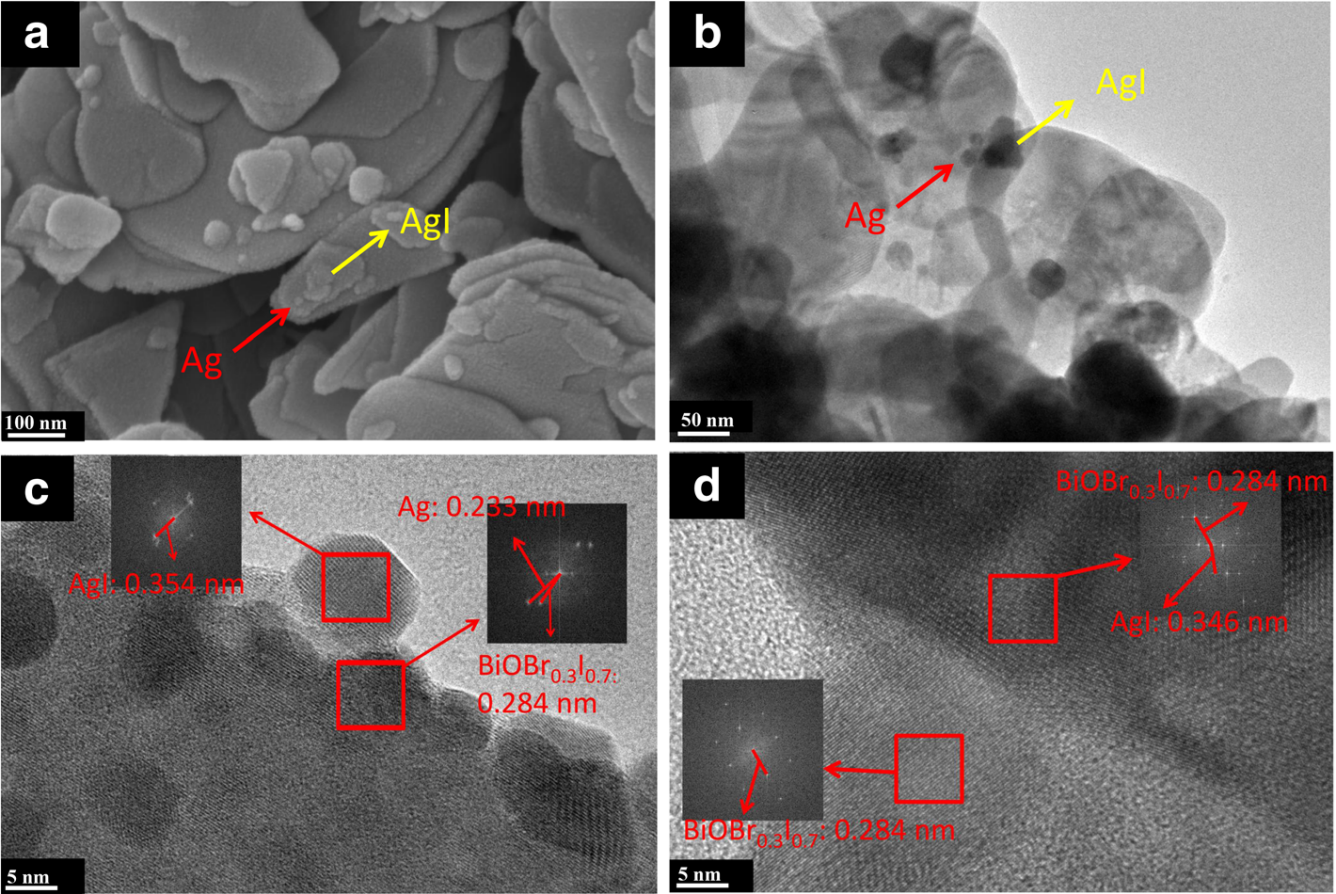
SEM images of BBA-3(**a**), TEM images of BBA-3 (**b**), HRTEM and FFT images of BAA-3 (**c**), and BiOBr_0.3_I_0.7_/AgI (**d**)

The morphology and microstructure of the samples were examined by SEM and TEM. The nanoplate structure with a size of 200 to 600 nm is related to BiOBr_0.3_I_0.7_ [13]. It can be observed that the small particle, approximately 10 nm in size, and the plate-like structure were formed in situ on the surface of BiOBr_0.3_I_0.7_, which can be related to Ag and AgI (Fig. 2a). To further investigate the morphology of BAA-*x*, TEM and HRTEM images were recorded, as well as Fourier transform infrared (FFT) images. It is clear that AgI and Ag nanoparticles were formed in situ on the surface of BiOBr_0.3_I_0.7,_ which is in agreement with the SEM result (Fig. 2b). The lattice of Ag (0.233 nm), AgI (approximately 0.350 nm), and BiOBr_0.3_I_0.7_ (approximately 0.285 nm) can be observed (Fig. 2c), indicating that Ag nanoparticles were formed in situ on the surface of the BiOBr_0.3_I_0.7_, making contact with BiOBr_0.3_I_0.7_ and AgI. Figure 2d shows the structure of BiOBr_0.3_I_0.7_/AgI and the junction between BiOBr_0.3_I_0.7_ and AgI; no Ag particle was observed. According to the SEM and TEM results, the BAA-*x* Z-scheme photocatalyst was successfully synthesized via in situ precipitation and photo-reduction methods.

### Composition Analysis and Optical Properties

The chemical composition and chemical state of BAA-x were analyzed by XPS spectroscopy. From the XPS survey spectrum (Fig. 1b), Bi, Br, I, O, and Ag were observed for the BAA-*x* sample. The strongest peaks at 164 eV and 159 eV correspond to Bi 4f_5/2_ and Bi 4f_7/2_ (Fig. 1c). The Br peak can be resolved into two components: the peak at 69 eV belongs to Br 3d_3/2,_ while the peak at 68 eV belongs to Br 3d_5/2_ (Fig. 1d)_._ The I peaks at 630 eV and 619 eV can be attributed to I 3d_3/2_ and I 3d_5/2,_ respectively (Fig. 1e). The Ag 3d peaks can be separated as Ag^+^ peaks and Ag^0^ peaks. The strong peaks at 374.2 eV and 368.2 eV can be assigned to Ag^+^ in BBA-*x*. The weak peaks at 373.6 eV and 367.6 eV can be assigned to Ag^0^ in BBA-*x*, indicating the existence of metallic Ag nanoparticles on the surface of the BAA-*x* sample (Fig. 1f).

The actual composition of the as-prepared samples was determined by SEM-EDS analysis. As shown in Table 1, the BiOBr_0.3_I_0.7_/AgI sample contains approximately 2.79% AgI, and the ratio of the rest of the elemental I to the elemental Br is approximately 7/3, implying that the deposited AgI does not change the element composition of BiOBr_0.3_I_0.7_. After light irradiation, the Ag element content in BAA-3 is higher than that in BiOBr_0.3_I_0.7_/AgI, indicating that metallic Ag is formed by in situ photo-reduction. The actual AgI and Ag contents of the BAA-*x* samples were calculated from SEM-EDS data (Table 1). The AgI content in the BAA-*x* sample was calculated from the Bi element content based on the Bi/I ratio in BiOBr_0.3_I_0.7_. The total Ag element content can be divided into two parts: AgI and metallic Ag; thus, the metallic Ag content can be calculated by the total Ag element content and AgI content. Metallic Ag can be found from all the BAA-*x* samples, indicating that Ag nanoparticles can be reduced by photo irradiation.

**Table 1:** EDS result of BAA-*x* samples and BiOBr_0.3_I_0.7_/AgI

Sample	Atom content (at. %)				AgI (%)	Metallic Ag (%)
	Bi	I	Br	Ag		
BAA-1	49.51	35.23	13.37	1.87	0.573	1.297
BAA-2	47.08	34.62	15.5	2.81	1.664	1.146
BAA-3	47.36	34.37	12.91	5.35	1.218	4.132
BAA-4	46.44	33.47	14.68	5.41	0.962	4.448
BiOBr_0.3_I_0.7_/AgI	50.75	33.4	13.06	2.79	2.79	0

**Fig. 3:**
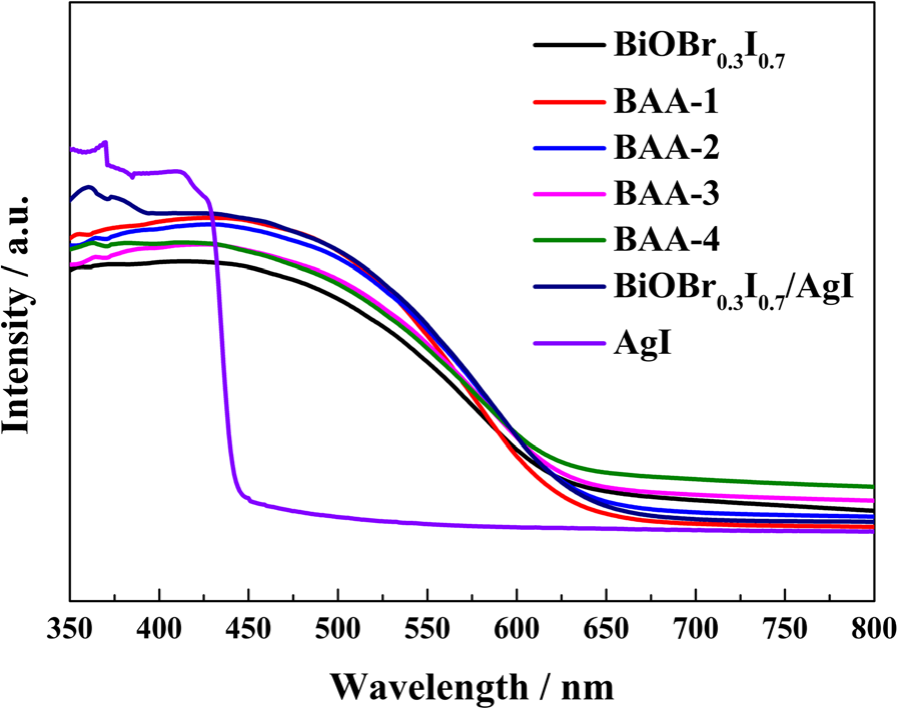
UV–vis DRS spectra of BiOBr_0.3_I_0.7_, BAA-*x*, and BiOBr_0.3_I_0.7_/AgI

Figure 3 shows the UV−visible diffuse reflectance spectroscopy (DRS) results of the obtained photocatalysts and AgI. AgI has an absorption edge at approximately 450 nm, while BiOBr_0.3_I_0.7_ has a broader absorption in the visible region from 400 to 650 nm. All BAA-*x* samples have similar absorption regions as BiOBr_0.3_I_0.7,_ while BAA-*x* exhibits stronger visible light absorption from 400 to 575 nm. As the Ag element content increases in BAA-*x*, the visible light absorption ability slightly decreases. According to previous reports, samples with a surface plasmon resonance (SPR) effect of Ag exhibit a noticeable increasing absorption band in the visible light range [25–27]. However, with the increase in Ag element content, BAA-*x* samples do not show an absorption band that could assigned to the SPR effect of Ag, indicating that the SPR effect of Ag is insignificant in the BAA-*x* samples [28].

The band gap potential of BiOBr_0.3_I_0.7_ and AgI was calculated by the Kubelka–Munk method based on the UV–vis DRS spectra, which are 1.61 eV and 2.83 eV for BiOBr_0.3_I_0.7_ and AgI, respectively. The band structures of BiOBr_0.3_I_0.7_ and AgI were calculated by the following empirical formulas.

1 $$ {E}_{\mathrm{VB}}=\chi -{E}_{\mathrm{e}}+0.5{E}_{\mathrm{g}} $$

2 $$ {E}_{\mathrm{CB}}={E}_{\mathrm{v}}-{E}_{\mathrm{g}} $$

where *E*_g_ is the band gap potential, *E*_VB_ is the valence band potential, *E*_CB_ is the conduction band potential, *E*_e_ is the energy of free electrons on the hydrogen scale, which is approximately 4.5 eV, and *χ* is the absolute electronegativity of the semiconductor, expressed as the geometric mean of the absolute electronegativity of the constituent atoms. Thus, the *E*_VB_ of BiOBr_0.3_I_0.7_ and AgI were calculated to be 2.71 eV and 2.52 eV vs. normal hydrogen electrode (NHE) and their corresponding *E*_CB_ are 1.10 eV and − 0.31 eV vs. NHE, respectively, which are in agreement with previous reports [13, 21].

### Photocatalytic Activity and Mechanism Experiments

**Fig. 4:**
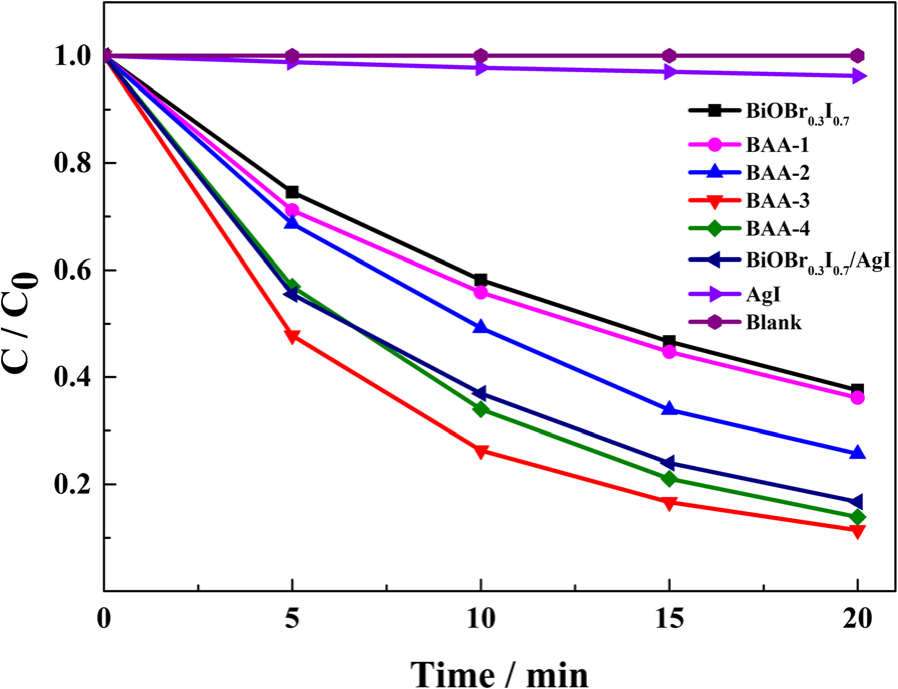
Photocatalytic activities of MO degradation presented by different samples

The photocatalytic activity of the prepared samples was evaluated by degradation of MO under visible light irradiation. Based on the blank (in the absence of any catalyst) experiment, the self-photolysis of MO under visible light irradiation can be ignored. As shown in Fig. 4, BAA-*x* exhibited superior photocatalytic activity compared with BiOBr_0.3_I_0.7_ and pure AgI. With the increase in Ag element content, the photocatalytic activity of BAA-*x* increased first and then decreased. This tendency matches with the previous reports of noble metal decorated semiconductors [28–31]. According to the EDS result and photocatalytic activity experiments, with a low added Ag element ratio (5%), the low amount of AgI cannot construct an efficient Z-scheme system to facilitate separation of the photo-generated carrier. When the added Ag element ratio was increased to 15%, BAA-3 exhibited the highest photocatalytic activity among the BAA-*x* photocatalysts, which degraded approximately 89% of MO within 20 min under visible light irradiation. This result indicated that the charge separation is more efficient for increased amounts of Ag nanoparticles. After increasing the added Ag element ratio to 20%, the photocatalytic activity of BAA-4 was depressed. The reason may be that the low AgI content led to excess Ag nanoparticles on the surface. Then, the excess Ag nanoparticles may have accumulated electrons, which attracted photo-generated holes, leading to the interfacial electron–hole recombination. BAA-3 exhibits higher photocatalytic activity than BiOBr_0.3_I_0.7_/AgI with the same elemental Ag content, demonstrating that the formation of Ag nanoparticles can improve the photocatalytic activity of BAA-*x*. Based on the above discussion, a conclusion can be drawn that BAA-*x* has a higher redox capability than BiOBr_0.3_I_0.7_, demonstrating that the fabrication of a Z-scheme photocatalytic system between BiOBr_0.3_I_0.7_ and AgI is an efficient strategy to improve the photocatalytic activity and the redox capability of BiOBr_0.3_I_0.7_.

**Fig. 5:**
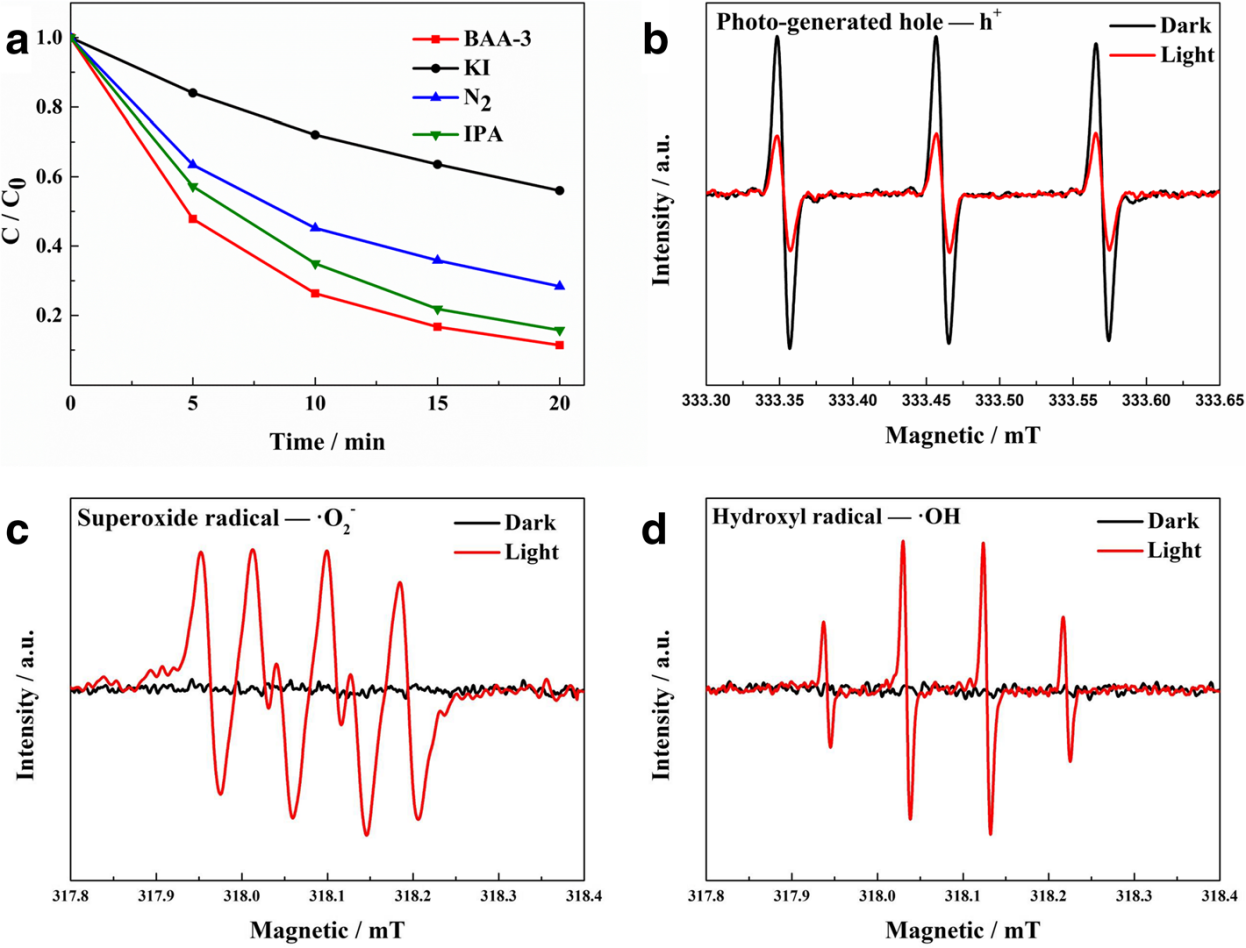
Results from the radical trapping experiment in the presence of BAA-15 (**a**) and the EPR spectra of the photo-generated hole (**b**), ·O_2_^−^ radical (**c**), and ·OH radical (**d**)

**Fig. 6:**
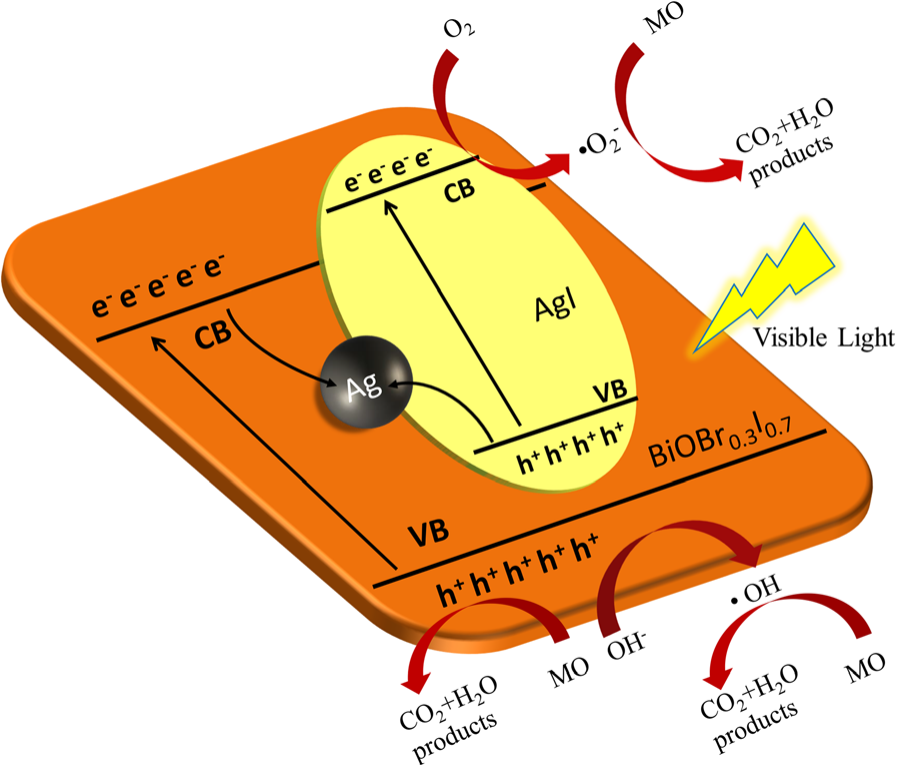
Schematic of the mechanism of the photodegradation of MO by BAA-*x*

In order to identify which radical species is the major active species in the photocatalytic process, radical trapping experiments and EPR measurements were carried out to examine the photocatalytic mechanism of BAA-*x*. In the radical trapping experiments, tertiary butanol (t-BuOH), N_2_, and potassium iodide (KI) were added as scavengers for hydroxyl radicals (·OH), superoxide radicals (·O_2_^−^), and holes (h^+^), respectively. KI and N_2_ have an obvious inhibition effect on the photocatalytic activity of BAA-3 (Fig. 5a), indicating that h^+^ and ·O_2_^−^ are the dominant active species, and h^+^ is the main active species in photocatalytic degradation. The photocatalytic activity of BAA-3 is slightly inhibited by the addition t-BuOH, indicating that a low amount of ·OH was formed during the photocatalytic process. Meanwhile, from EPR experiments (Fig. 5b–d), when BAA-3 is under visible light irradiation, ·OH and ·O_2_^−^ signals can be detected and the h^+^ scavenger 2,2,6,6-tetramethylpiperidine (TEMP) signal is decreased, which is in agreement with the radical trapping experiment. According to the mechanism and band structure study, the possible Z-scheme photocatalytic mechanism of BAA-*x* is shown in Fig. 6. Under visible light irradiation, the electrons in the valence band (VB) of BiOBr_0.3_I_0.7_ and AgI are both excited to their conduction band (CB); after that, the photo-generated electrons in the CB bottom of BiOBr_0.3_I_0.7_ move to Ag nanoparticles and then continue to shift to the VB top of AgI, recombining with the photo-generated holes there. The remaining photo-generated electrons in the CB of AgI (− 0.31 eV) with more negative potential exhibit strong reduction ability and react with O_2_ (O_2_/ ·O_2_^−^ = − 0.046 eV, vs. NHE) [21], allowing ·O_2_^−^ to degrade MO. Meanwhile, the photo-generated holes (2.71 eV) in the VB of BiOBr_0.3_I_0.7_ display strong oxidation ability to directly degrade MO, and according to the radical trapping experiments, a small number of photo-generated holes react with H_2_O to generate ·OH (·OH/OH − =1.99 eV, vs. NHE) [32], which can further degrade MO.

## Conclusion

Novel all-solid-state Z-scheme BAA-*x* photocatalysts were prepared via facile in situ precipitation and photo-reduction methods. BAA-*x* exhibited excellent photocatalytic activity for the degradation of MO compared to BiOBr_0.3_I_0.7_ under visible-light irradiation. Enhancement of the photocatalytic activity is probably related to the special Z-scheme charge-carrier migration pathway, leading to the efficient separation of the photo-generated electron–hole pairs and sustained high redox capability. The optimal added Ag element molar ratio of BAA-*x* is 15%. Mechanistic experiments indicated that ·O_2_^−^ and h^+^ are the active radicals and that a low amount of ·OH is generated during photocatalytic degradation. Based on the above study, BAA-*x* shows potential for practical application in environmental purification of organic pollutants.

## Abbreviations

AM 1.5: Air mass 1.5
CB: Conduction band
EDS: Energy-dispersive spectroscopy
EPR: Electron paramagnetic resonance
FFT: Fourier transform infrared
HRTEM: High-resolution transmission electron microscopy
MO: Methyl orange
NHE: Normal hydrogen electrode
SEM: Scanning electron microscopy
SPR: Surface plasmon resonance
t-BuOH: Tertiary butanol
TEM: Transmission electron microscopy
UV–Vis DRS: Ultraviolet-visible diffuse reflectance spectroscopy
VB: Valence band
XPS: X-ray photoelectron spectroscopy
XRD: X-ray diffraction
